# Dr. Thomson's Report on Respiration and Animal Heat

**Published:** 1842-07

**Authors:** Robert Dundas Thomson


### Report on the present state of our Knowledge respecting Respiration and Animal Heat, and on Professor Liebig's new Chemico-Physiological Theory. By Robert Dundas Thomson, M.D.

Before stating Liebig's views it seems necessary to say a few words on those previously entertained. We shall suppose our readers acquainted with the earlier history of the progressive discoveries in this department of chemistry. If they are, they must be as much surprised as we are at some late pretensions of M. Dumas to originality. "The fact," he says, "of the absorption of oxygen in the act of respiration has been long known, as well as its transformation into water and carbonic acid; but a more recent idea is that the oxygen does not burn the hydrogen and carbon at one effort. It passes before the completion of this simple result, through a series of successive combinations, of which the carbonic acid and water are only the termination and not the point of departure, as had been previously thought," (Dumas, Cours de 1839.) In another passage he fairly claims the theory of Lagrange and Hassenfratz; and to prevent mistakes we quote his own words: "Nous avons professé que le sang s'arterialise dans le poumon, sans produire de chaleur, et qu'il respire réellement dans les capillaires du corps tout entier en produisant l'acide carbonique, l'eau et la chaleur. Cette théorie est exposée dans une thèse du 15 Juillet, 1839. Il serait facile de prouver qu'elle a été professée en 1838." (Ann. de Chim. Jan. 1842.) Now this is nothing more than a brief epitome of the theory of Lagrange and Hassenfratz; and how such a distinguished chemist as Dumas could have claimed it as peculiar to himself, is one of those mysteries which require explanation.

Dumas has asked "to whom belongs the true theory of animal heat?" "This theory belongs incontestably," he answers "to Laplace and Lavoisier." Unquestionably the theory received much of its development from the labours of these French philosophers. But it should never be forgotten that Dr. Black was the first to point out the source of the heat. It is the more necessary to attend to this position, because neither Lavoisier nor his successor Dumas ever alludes to the name of our illustrious countryman. Even Despretz has been undervalued by Dumas in estimating the claim of different chemists to the development of the theory of respiration. The following results are deducible from the experiments of Despretz:

1. It appears that during the combustion of an avoirdupois pound of carbon the quantity of heat evolved is sufficient to melt 104·2 lbs. of ice. Now if the latent heat of water be 140°, 104·2 lbs. of ice will require 14588° of heat to melt it; or in other words the heat evolved during the combustion of 1 lb. of carbon would heat 1 lb. of water 14·588°. 2. The oxygen required to consume 1 lb. of carbon amounts to 2⅗ lbs., which is equivalent to 55·082 cubic inches, at the temperature of 60°. This oxygen combines with carbon, and is converted into its own volume of carbonic acid gas. 3. The 55·082 cubic inches of oxygen when converted into carbonic acid give out 14·588° of heat. Hence every 3¾ c. in. of oxygen when converted into carbonic acid give out 1° of heat. 4. Despretz farther showed that when 1 lb. of hydrogen is burned a quantity of heat is evolved capable of melting 315·2 lbs. of ice, or the heat evolved would heat 1 lb. of water 44·128°. But for this combustion 8 lbs. of oxygen are required. Now 8 lbs. of oxygen are equivalent to 165·246 c. i. Hence every 3¾ c. i. of oxygen, when they combine with hydrogen, evolve 1° of heat.

From these results we draw the conclusion that the heat evolved during the combustion of carbon and hydrogen is proportional to the quantity of oxygen gas consumed. But a somewhat different result takes place in the combustion of pulmonary respiration, according to the researches of the same laborious philosopher. For if we reckon the animal heat evolved in his experiments at 100° then the portion of it due to the combination of the oxygen of the atmosphere with carbon and hydrogen during the circulation of the blood through the body will be 82°. Consequently 18° or about ⅕ must be owing to other processes not yet detected.

Dumas objects to the experiments of Despretz, that an animal when surrounded by a cold fluid has its temperature lowered, and that therefore results obtained under such circumstances cannot be relied upon as strict expressions of truth.

Admitting then that the oxygen inspired by the lungs combines gradually with carbon in its progress through the system, and that carbonic acid is the result of this chemical union, it is natural to inquire if the carbonic acid is simply absorbed by the blood, or if it is transferred in any other form through the circulation. Mitscherlich, Gmelin, and Tiedemann have proposed a theory which they think is adequate to account for the disposal of the carbonic acid. The grounds of their theory are that acetic and lactic acids exist in a free or combined form in most of the secretions, and also in the blood. These acids they suppose to be generated in the body. They have determined likewise, that venous blood contains 12·3 per cent. of alkaline carbonates, while arterial blood contains only 8·3 per cent. They conceive that the air acts upon the blood during respiration so as to produce acetic acid, which, meeting with the carbonate of the venous blood, sets free the carbonic acid and combines with the alkaline base; and that the oxygen of the inspired air unites in part directly with carbon and hydrogen, producing carbonic acid and water, and in part enters into combination with the organic compounds contained in the blood, (Müller's Phys. by Baly, p. 343.)

After such a perspicuous announcement of this ingenious theory, which has now been before the public of this country for five years, it is rather startling to meet with the following statement brought forward by Dumas as the enunciation of a theory peculiar to himself. "The oxygen absorbed serves to burn lactate of soda, and in general salts of soda, which it changes into carbonate of soda. The lactic acid transforms the latter into lactate, and disengages carbonic acid. This lactic acid proceeds from the saccharine or amylaceous aliment." (Ann. de Chim. iv. 3d ser. 142.)

The only difference in the two statements is with respect to the origin of the lactic acid; and this could not have been otherwise, because it is only since the announcement of the German philosophers, that the derivation of lactic acid from sugar and starch has been clearly demonstrated. We confess that we have some difficulty in accounting for the conduct of Dumas. In general he is better acquainted with the history of his science than any of his countrymen. So it might be said was Lavoisier, and yet he never acknowledged in his theory of combustion, the obligations he lay under to the discoveries of Black. Nay, he never noticed the discovery of oxygen by Priestley, (who had, in the simplicity of his heart, communicated the discovery to him,) but actually spoke of the new element as if it had been discriminated by himself alone.

We now proceed to notice some of the recent labours of Professor Liebig in this most interesting department of physiology; we are happy in being enabled to give an accurate outline of his new chemico-physiological theory, derived from the most authentic sources. The professor is now publishing a work on the subject, from which we anticipate an impulse calculated to lead to the most momentous discoveries in medical science. This work will soon appear in English as well as in German.

*The source of animal heat.* Absorption of nourishment and of oxygen are the first conditions for the sustenance of animal life. During every moment of his existence man imbibes oxygen by his respiratory organs. According to Menzies, a human adult takes up from the atmosphere 850 lbs. of oxygen during the year; and yet at the end of that period his weight remains perfectly unchanged, or only differs perhaps by a few ounces. What becomes of this enormous quantity of oxygen which is thus consumed, is a natural subject of inquiry. No part of this oxygen remains in the body, but it is again discharged under the form of a compound with carbon or hydrogen. The carbon and hydrogen of certain constituents of the body have united with the oxygen absorbed through the lungs and skin, and are expelled in the shape of carbonic acid and water. If we consider, with Lavoisier and Seguin, the quantity of oxygen consumed by an adult daily to be 30½ oz.=15,661 Fr. grains, and reckon the quantity of blood 24 lbs. of which 80 per cent. is water, it follows that to turn the whole of the carbon and hydrogen of the blood into carbonic acid and water would require 66040 Fr. grs. or upwards of 120 oz. of oxygen; and this operation would be completed in 4 days and 5 hours. The food supplies the carbon and hydrogen required in this process. From a carefully conducted set of experiments made upon 856 soldiers, Liebig infers that an adult takes up daily 13 oz. of carbon. This was determined by weighing the food and fæces daily for a month. The fæces amounted to 7 oz. daily: they contained 75 per cent. of water, and the dry residue 42¼ per cent. carbon, 13·15 per cent. of ashes; 100 parts of fresh fæces, therefore, contain 11·31 carbon; or very nearly as much as an equal quantity of fresh meat. The 13 oz. of carbon which are daily taken into the system are discharged by the skin and lungs in the form of carbonic acid. For their conversion into carbonic acid these 13 oz. require 34⅔ oz. of oxygen. According to the experiments of Boussingault, a horse takes up, in 24 hours, 74½ oz. of carbon, and a milch cow 66¼. To convert these into carbonic acid the horse requires from 13 to 14 lbs.; and the cow from 11 to 12 lbs. of oxygen. Now as none of the oxygen is thrown off from the system in any other form than that of carbonic acid and water, and as the carbon and water are derived from the food, it follows that the quantity of nourishment required for the support of the system is in direct proportion to the quantity of oxygen taken up. Two animals which consume in equal periods of time unequal quantities of oxygen by the skin and lungs, require in the same proportion an unequal weight of food. In equal periods the consumption of oxygen depending on the number of respirations, it is clear that in one and the same animal the quantity of food digested varies according to the strength and number of the respirations. A child whose respirations are more frequent must require proportionally more nourishment than an adult, and can less easily bear hunger. A bird dies from want of food on the third day. The serpent which when placed for an hour under a receiver consumes scarcely so much oxygen as to enable the resulting carbonic acid to be detected, lives for three months and even longer without food. In a state of rest the number of respirations is less than when the body is actively employed. The quantity of food required in both circumstances must bear the same proportion. An excess of food and a deficiency of inhaled oxygen (or exercise), as well as great exercise (which enforces a greater imbibition of nourishment) and weak digestive organs, are incompatible with each other.

The quantity of oxygen, according to the view of Liebig, which an animal absorbs in the lungs is not altogether dependent on the number of respirations, but it is closely connected with the temperature of the inspired air. The cavity of the chest of an animal remains always possessed of the same capacity; at each respiration the same volume of gas enters. But the weight of this volume, and therefore the weight of the oxygen contained in it, is not always equal. When an adult breathes 46,037 cubic inches of oxygen of the temperature 77°, the weight of the oxygen amounts to 30½ oz. But if the same volume of oxygen be breathed at the temperature of 32°, the weight of oxygen will be 32¾ oz. In summer and in winter, at the Pole and at the Equator, we breathe the same bulk of air; and while in summer we inhale in an equal number of inspirations 29½ ounces, the quantity of oxygen inhaled at 32° is 32¾ ounces; in Sicily (at 95°) 26¾ ounces; at 14°, 33¾. At a lower temperature we expire more carbon than at a higher temperature, and we must in the same proportion employ more or less carbon in our food; in Sweden more than in Sicily; and in Germany an eighth part more in winter than in summer. Even in comparing equal quantities of food in cold and warm countries, we find the quantity of carbon to be very unequal. The fruits which the inhabitants of the tropics employ contain in the fresh state 12 per cent. of carbon; while the fat and oil of the Esquimaux contain from 66 to 80 per cent. of carbon. There is no great difficulty in practising moderation in warm countries or in enduring hunger for a considerable period under the equator; but cold and hunger soon produce death. According to Liebig, therefore, the reciprocal action of the constituents of the food and of the oxygen disseminated through the circulation in the body is the source of animal heat.

All living beings whose existence depends upon their absorption of oxygen, are dependent for one source of their animal heat on the atmosphere which surrounds them. This truth applies to all animals. It extends to the germinating seed, to the flowers of plants, and to fruits which are attaining maturity. Heat is only evolved in those parts of animals to which the arterial blood and the oxygen imbibed by respiration are distributed. Hair, wool, and feathers possess no peculiar temperature. The higher temperature of animals, or in other words the greater extrication of heat in animals, is always the consequence of a combination between a combustible substance and oxygen. For in whatever form the carbon combines with the oxygen, the act of combination cannot take place without the evolution of heat. Whether the union is effected slowly or with rapidity, the resulting heat is ultimately exactly the same. The carbon of the food which is converted into carbonic acid in the bodies of animals must evolve the same amount of heat as if it were burned in the air or in oxygen gas. Animals which breathe rapidly, and therefore consume much oxygen, possess a higher temperature than those which breathe more slowly. The temperature of a child is 102½°, that of an adult 98 to 100°. The heat of a bird is 106 to 109°, and is greater than that of mammiferous animals, which have a temperature varying 99 to 106°; or than that of fishes, whose heat exceeds that of the surrounding water in which it is immersed by only a degree or two. All animals are warm-blooded, but it is only in those which respire by lungs that we find their peculiar temperature completely independent of the temperature of the surrounding media. An animal is a heated body which is acted on by the surrounding atmosphere, as all heated bodies are; it imbibes heat when the surrounding atmosphere is hotter than itself, and it gives out heat when the atmosphere is colder. It hence follows that at the Pole, with the temperature below zero, the loss of heat must be much more rapid than at the equator. Yet the blood of the Esquimaux and that of the inhabitants of the tropics possesses the same temperature—a clear proof that the heat must be renewed more quickly in winter than in summer, and more rapidly at the Pole than at the Equator. In different climates it is obvious that the quantity of oxygen consumed in respiration must depend on the temperature of the external air. With the loss of heat by cooling, the quantity of respired oxygen increases. The carbon and hydrogen necessary for combination with the oxygen must vary in a similar proportion. It is clear that the compensating heat will be produced by the reciprocal action of the constituents of the food which combine with the inspired oxygen.

The animal body may in some respects be compared to an oven which we supply with combustible materials. In like manner, whatever forms the food gradually assumes in the body, and whatever transformations it may undergo, the last alteration is a conversion of its carbon into carbonic acid, and its hydrogen into water; while the azote and unburned carbon are excreted in the urine and fæces. To retain the temperature of an oven constant, it is necessary to supply combustible materials in unequal quantities, according to the changes of the exterior temperature. In relation to animals the food is the combustible matter; by the due access of oxygen and the oxidation of the food, heat is evolved. In winter, when by movement in colder air, the quantity of the inspired oxygen increases, the necessity for nutriment rich in carbon and hydrogen varies in the same proportion; and in compensation for this necessity we obtain the most perfect protection against the severest cold. A hungry man shivers; and the animals of prey of northern climates are more voracious than those of southern countries. Our clothing is merely an equivalent for food; in proportion to the warmth of our clothing the necessity for eating diminishes. The quantity of food, therefore, which is used depends on the number of respirations, on the temperature of the air which we breathe, and on the quantity of heat which we give out to the air. No isolated influence can alter this law. The European, when residing in tropical countries, endeavours in vain to stimulate his organs with powerful condiments to imitate the appetite which he indulged in at home. English patients, whose digestive organs are out of order, are sent to southern countries, where the quantity of inspired oxygen is diminished and the nourishment of the body proceeds with less labour to the organs of assimilation. In summer the most prevalent complaints in Germany are liver-diseases (carbonaceous diseases;) in winter lung-diseases, (oxygenous diseases.)

The whole process of respiration is clearly exhibited when we take a view of the condition of a man or animal under abstinence from all food. There will be, as before, oxygen abstracted from the air, and carbonic acid and water expired, because the number of respirations remain unaltered. We know with precision from whence the carbon and hydrogen emanate; and with the continuance of the abstinence we see the carbon and hydrogen of the body diminishing. The first effect of hunger is the disappearance of the fat. This fat can be detected neither in the scanty fæces nor in the urine; its carbon and hydrogen are thrown off by the skin and lungs in the form of a compound with oxygen. It is obvious that these constituents have served for the purposes of respiration. Every day 32½ oz. of oxygen are inspired, and these must remove their equivalents of carbon to form carbonic acid. When this combination ceases to go on respiration terminates—death has taken place. The time required for starving an animal to death depends on its fatness, on the state of its activity, on the temperature of the air, and lastly on the presence or absence of water.

In all chronic diseases death occurs from the same cause—from the action of the atmosphere. When the materials fail which are destined for the sustenance of respiration in the organism; when the organs of the sick refuse to perform their functions; when they lose their capacity to transfer the food into that state necessary for its combination with the oxygen of the air—then their own substance, the fat, brain, muscles, and nerves will be attacked. The peculiar cause of death is, in this case, the process of respiration, the influence of the atmosphere.

*Oxidation, not the nerves, the cause of animal heat.* None will deny the importance of the nervous system in the process of respiration, for no change of state can occur in the animal economy without the influence of the nerves. By their action the intestines bring the combustible materials into a condition fit for their combination with oxygen; and in the absence of their functions the whole act of the imbibition of oxygen must assume another form. Yet it cannot be doubted that the influence of the nerves in respiration, and in the production of animal heat, has been much overrated.* Liebig even goes so far as to declare that the idea of the evolution of animal heat by the action of the nerves is an absurdity; for if we exclude chemical action, or changes in the arrangement of the elementary particles as a condition of nervous agency, it means nothing else than to derive the presence of motion, the manifestation of a power, from nothing. But no power can come of nothing.†

That the quantity of heat evolved by the combustion of 13·8 oz. of carbon is amply sufficient to account for the temperature of the human body, may be readily gathered from the employment of numbers. An oz. of carbon burned would evolve 14207°, and 13·8 oz. would therefore give out 197477·3°. This would suffice to boil 136·8 lbs. of water at 32°, or to convert 24 lbs. of water at 98° into vapour. If we consider then the quantity of water vaporized through the skin to be in 24 hours 48 oz. (3lbs.), there will remain 146380° of heat

* Some have endeavoured to discover a source of animal heat in the contraction of the muscles; forgetting that in their expansion an equal quantity of heat was absorbed. All genuine sources of heat in the body must probably be referred to chemical action.

† The consideration of power is most important. Heat, it is said, may be produced by *friction*. This mode of expression keeps out of view the cause of friction; friction being only an effect of power.

which are dissipated by radiation, by heating the expired air, and by the excrementitious matters. Liebig considers that experiments made upon the quantity of carbonic acid expired by the usual tests are of no value; because so much depends upon the density and temperature of the air, and other circumstances, that it is impossible to calculate accurately. The degree of motion, labour, or exercise, the amount and quality of the food, the comparative warmth of the clothing, and also the time when the food is taken, are important elements in this mode of investigation. Liebig prefers the method already referred to, by determining the composition of the food and that of the excretions. Prisoners in the house of correction at Marienschloss, where labour is enforced, consume 10½ oz. of carbon daily, while in the house of arrest at Giessen, close by the Hessian laboratory, the consumption of carbon is only 8½ oz. The quantity consumed by soldiers engaged in healthy exercise, we have already stated to be 13·8 oz., while in a family of five adults and four children, the average daily consumption of carbon for each was 9½ ounces.

*The constituents of the blood exist in plants.* From the unwearied researches of Liebig and his pupils, the important fact has forced itself upon our acceptance, that where vegetable life ends, animal life begins. Plants prepare vegetable fibrin, vegetable albumen, and vegetable casein for the nourishment of animals. These are swallowed by animals as food, and are conveyed into the blood without even modification. The albumen and fibrin of the blood existed in plants under the denomination of vegetable fibrin and albumen; they are absolutely identical. Hence Liebig observes, "we may say that the animal organism merely gives to blood its form, and that it is incapable of creating blood out of other substances, which do not already contain the chief constituents of that fluid." The three bodies, which we have described as serving the purpose of supplying substance to the body, all contain azote. What then, it may be asked, is the use of starch, sugar, gum, pectin, &c., which contain no azote, and do not enter into the formation of the solids of the system? Graminivorous animals must be supplied with one or other of these substances. If they are withheld, death rapidly ensues. The young of carnivorous animals are nourished in a somewhat similar manner to graminivorous animals; for milk contains only one azotized principle, *cusein*, while its other constituents are butter and sugar; but casein is identical in its composition with the albumen of peas, beans, and lentils. These vegetables are capable of producing animal blood; from them, therefore, the mother's blood may be formed, and from the blood is secreted the milk which serves as nourishment to the young. The utility then of the casein and similar compounds is sufficiently obvious; but that of the butter and sugar is not so easily detected. Liebig considers that they supply a certain amount of carbon and hydrogen to the azotized constituents of the food, or, in other words, that they afford a supply of carbon and hydrogen in excess, which is expended on the production of animal heat, and serves to protect the organism from the action of the oxygen of the atmosphere. He adduces several familiar instances in support of his position: the boa-constrictor, when a bird or rabbit is given to it, retains its original weight, consuming the flesh, blood, bones, and nerves of its victim, and, expelling a pure white substance like chalk, urate of ammonia, which contains for every equivalent of azote two of carbon; but the food which it had swallowed contained eight equivalents of carbon to one of azote. This immense consumption of carbon can only have taken place through the skin and lungs in the form of carbonic acid gas. Again, the excrement of lions consists chiefly of bone earth, with mere traces of compounds of carbon; their urine contains not urate of ammonia, but urea, in which the azote is to the carbon as one to one; but their food contained one of azote to eight of carbon. The whole of the excess of carbon has disappeared, in the process of respiration, as carbonic acid; and its accompanying hydrogen in the form of water. Now, if the flesh which constituted the food of the lion, had been burned in a furnace, we should have obtained nearly the same results; the only difference would have been in the form of the azotic compound. The azote would have appeared in the state of carbonate of ammonia, while the remaining carbon and hydrogen would have been converted into carbonic acid and water; precisely as in the process of respiration, the combustible parts would have assumed the form of ashes, and the unconsumed carbon would have been given out as soot or lampblack. Now, in the animal the solid fæces are only the incombustible or imperfectly burned parts of the food.

*The bile supplies carbon to combine with oxygen.* Although the oxidized products given off by respiration correspond with the carbon and hydrogen taken into the system in the form of food, and with the nitrogen in the shape of a compound containing the same elements as carbonate of ammonia, we are not to conclude that the only use of the food is to produce carbonic acid, water, urea, and uric acid; on the contrary, Liebig infers that the tissues are continually undergoing metamorphosis, that their transformation is the source of the excretions, while the blood deposits new matter in their place. The nitrogen of the exhausted or transformed organs appears in the bladder in the form of urine; the carbon of the transformed tissues appears in the gall-bladder in the shape of bile, a compound of soda (choleate of soda), and these transformations produce animal heat. But the bile is not an excretion, neither is it merely a stimulant to the intestinal canal. What is it, then? Chemical research shows us that it is taken up by the absorbents even of the rectum; when injected by the rectum, the whole of the bile disappears with the injected fluid; none whatever can be detected in the fæces. Bile, therefore, differs from urine in this respect, that the urea and uric acid of the urine are expelled for ever as useless to the system, while the carbon of the bile serves as an ulterior combustible material. The food of the carnivora is converted into blood which is destined for the reproduction of organized tissues; and by means of the circulation, a current of oxygen is conveyed to every part of the body. The globules of the blood, it can be demonstrated, have no share in supplying nutrition to the system; but they serve to transport oxygen through the sanguineous canals, and give it up in order to combine with carbon and hydrogen in the capillary vessels; for here the current of oxygen meets with the compounds produced by the transformation of the tissues, and combines with their carbon to form carbonic acid, and with their hydrogen to form water. Every portion of these substances which escapes this oxidating process is sent back into the circulation in the form of the bile, which by degrees gradually disappears. In carnivorous animals, all the food is capable of forming blood. Their excrements, with the exception of the urine, contain only inorganic substances; no bile and no soda are present; but although soda is an important constituent of the bile, we do not find it in the fæces except in the shape of common salt and sulphate of soda, which exist in all the animal fluids. The soda of the bile must, therefore, have returned from the alimentary canal into the organism. The bile affords a plentiful source of carbon, much more fruitful perhaps than might appear at a superficial glance: it is estimated that a man secretes daily from 17 to 24 oz. of bile, a large dog 36 oz., a horse 37 lbs., or 592 oz., of which 59·2 oz. are solid matter; but no portion whatever of the excrement is bile. During the digestive process, therefore, the whole of the bile is returned into the blood, the soda reappears in the newly-formed blood, and, lastly, we find it in the urine in the form of phosphate, carbonate, and hippurate of soda. A similar observation applies to human bile. Man secretes from 9640 to 11520 grs. of bile daily; but only $\frac{1}{80}$ of a substance in the slightest degree resembling bile can be detected in the fæces.

From these data and the considerations previously stated, the remarkable conclusion is necessarily drawn, that all the carbon of the food is ultimately given out in the form of carbonic acid; it further appears that the carbon of the carbonic acid given off with that of the urine, the nitrogen of the urine and the hydrogen given off as ammonia and water; these elements, taken together, must be exactly equal in weight to the carbon, nitrogen, and hydrogen, of the transformed tissues, and also to the carbon, nitrogen, and hydrogen of the food. If this were not the case; the animal would alter in its size, and so it happens with the young of animals. They increase appreciably every day—and how do they increase? In the young animal, the assimilative process is more energetic, more intense, than the process of transformation: the circulation is more rapid, the respirations are more frequent, and for equal bulks the oxygen consumed must be greater in the young than in the adult animal; but as the metamorphosis of organized parts proceeds more slowly, there would obviously be a deficiency of carbon and hydrogen to combine with the oxygen inspired. To obviate this difficulty, Infinite Wisdom has supplied to the young the carbon and hydrogen of the butter of milk, and the carbon of sugar, for the support of the respiratory system at an age when a greater resistance is opposed to the metamorphosis of existing tissues, or, in other terms, to the production of compounds which, in the adult, are formed in quantity amply sufficient for the purposes of respiration. The young animal receives the constituents of its blood in the casein of the milk, a transformation of existing tissues goes on, for bile and urine are secreted; the matter of the metamorphosed parts is given off in the form of urine of carbonic acid and water; but the butter and sugar of milk also disappear, and cannot be detected in the fæces. The butter and sugar of milk are given out in the form of carbonic acid and water; and their conversion into oxidized products furnishes the clearest proof, that far more oxygen is absorbed than is required to convert the carbon and hydrogen of the metamorphosed tissues into carbonic acid and water. These considerations, Liebig conceives, leave no room for doubt, that nature has added to the food of the young of carnivorous animals substances destitute of azote, which their organism cannot employ for nutrition, strictly so called, that is, for the production of blood; substances which may be entirely dispensed with in their adult state. In the young of carnivorous birds, the want of all motion is an obvious reason why there is a diminished waste in the organized parts; hence milk is not provided for them.

*Sugar, starch, and gum supply carbon to burn the excess of oxygen inspired.* In graminivorous animals, we find that substances possessing an identical composition with sugar of milk, are required for their existence. Starch, sugar, and gum, are all similar in their composition, as the following table shows.

| | | |
|---|---|---|
| Starch | 12 atoms carbon | and 10 atoms water. |
| Cane sugar | " | 1 |
| Gum | " | 4 |
| Sugar of milk | " | 2 |
| Grape sugar | " | 4 |

Starch is readily converted into sugar, and the change takes place on the ripening of fruit; unripe apples contain starch, but not a trace of it can be detected in their ripened state. Gum resembles starch in not being fermentible. Sugar of milk is in the same condition, but it may be fermented by exposing it to heat in contact with putrefying cheese: in this case it is, however, first converted into grape sugar. The function performed on the vital processes of graminivorous animals by these substances, is pointed out in a clear and convincing manner, when we consider the very small relative amount of carbon consumed by them in the azotized constituents of their food, which bears no proportion whatever to the oxygen absorbed by the skin and lungs. A horse, for example, in Germany may be kept in good condition by feeding him daily with 15 lbs. of hay and 4½ lbs. of oats: now, as hay contains 1½ per cent and oats 2·2 per cent. of azote, the horse receives no more daily than 4½ oz. of azote, corresponding to 8 lbs. of blood; but along with this azote that is combined with it in the form of fibrin and albumen, the animal receives only about 14¾ oz. of carbon; only 8 oz. of this can be employed to support respiration: for with the nitrogen expelled in the urine, there are combined in the form of urea 3 oz., and as hippuric acid $3\frac{1}{2}$ oz. of carbon. But man consumes daily about 14 oz. of carbon, and the horse, according to Boussingault, 79 oz. daily; hence it appears that in the azotized constituents of its food, the horse receives rather less than the fifth of the carbon necessary for its respiration. The remaining four-fifths must inevitably, therefore, be supplied by the starch, sugar, or gum, which constitute the complementary elements of nourishment.

It appears now obvious that in graminivorous animals, whose food contains such a small proportion relatively of the constituents of blood, the process of the transformation of existing tissues must proceed much less rapidly than in carnivorous animals, otherwise the vegetation of the globe would scarce be sufficient to maintain them. A nation of hunters eat large quantities of animal food and little else. When they are kept within certain limits, its members must increase but sparingly, because the carbon which they consume is supplied mostly by animals. These animals must reside within the boundaries of their nation, and must be supplied with their carbon from the plants growing within these limits. Can we wonder then at the depopulation of the Indian parts of North America? Fifteen lbs. of flesh contain no more carbon than 4 lbs. of starch. If the savage, therefore, with *one* animal and an *equal* amount of starch, could maintain life for a certain number of days, he would be compelled if confined to flesh alone, in order to procure the carbon necessary for respiration, to consume during the same period *five* such animals. How close then is the connexion between agriculture and the increase of the human species! The cultivation of our crops has ultimately no other object than the production, on the smallest possible space, of a maximum of those substances which are adapted for assimilation and respiration. When man is restricted to animal food, he respires like the carnivorous animals, at the expense of the matters produced by the transformation of the organized tissues, and in order to effect this he requires, like the lion and other beasts of prey, to undergo a vast amount of muscular exercise; he is compelled to expend a given amount of *power* merely to supply matter for respiration. Cultivation is the economy of power. Science teaches us the simplest method of obtaining the greatest effect at the smallest expenditure of power, and to produce with given means a maximum of power.

When we compare the capacity for increase of mass in the assimilative power of carnivorous and graminivorous animals, a most marked difference is at once observable. A spider sucks with great voracity the blood of the first fly, but is not excited or disturbed by a second or third. A cat will eat the first, or perhaps the second mouse presented to her, but even if she kills a third she does not devour it. But, on the contrary, a cow or sheep in the meadow continues to eat with little interruption from sunrise till sunset. Their systems possess not merely the power of supplying the waste created by the metamorphosis of the tissues, but of converting into organized tissue all the food they devour. The excess of blood produced is converted into cellular and muscular tissue; the graminivorous animal becomes fleshy and plump, while the flesh of the carnivorous animal is always tough and sinewy. The animal in this state eats and reposes merely for digestion. Want of exercise is however equivalent to a diminution in the consumption of oxygen, and the excess of carbon is deposited in the cellular tissue in the form of *fat*.

*Fat originates from starch, &c.* When the horse and ox are in their normal condition, their urine contains benzoic acid (with 14 equivalents of carbon), but when the animal is kept in the stall, hippuric acid appears (with 18 equivalents of carbon). Wild animals are never fat; while stall-fed animals are covered with fat. But when the latter are permitted to move freely in the air, or compelled to draw heavy loads, that is to consume more oxygen, the fat again disappears. It is hence evident that the formation of fat is the result of a want of due proportion between the food swallowed and the oxygen absorbed by the lungs and skin. A pig when fed with highly azotized food increases in flesh, but when fed with potatoes it becomes fat. From these and other facts it appears logical to infer that fat is derived from vegetable food. If we compare the composition of sugar of milk with starch, sugar, mutton, beef, and human fat, we find that in all of them the proportion of carbon to hydrogen is the same; that is, 45 to 6, and that they only differ in that of oxygen. From which it follows, that by the mere separation of oxygen, it is possible that sugar, starch, and gum, may pass into fat. The following formulæ exhibit distinctly the differences between fat and starch:

| | | | |
|---|---|---|---|
| Starch...... | $C_{12}$ | $H_{10}$ | $O_{10}$ |
| Fat........ | $C_{11}$ | $H_{10}$ | O |

The only distinction therefore is in the absence from the latter of one atom carbonic acid ($CO_2$) and 7 atoms oxygen. If further we remove from 3 equivalents of milk sugar, 4 equivalents of water, and 31 of oxygen, we have remaining a formula which exactly represents the composition of cholesterine, the fat of the bile. But from whatever source fat may be formed, it is certain that its production can only take place in one way, viz., by a separation of oxygen from the elements of the food. The formation therefore of fat depends on a deficiency of oxygen, but in the production of fat a new source of oxygen is developed, *a new cause of animal heat.* The oxygen evolved when the fat is deposited must combine with carbon or hydrogen from some other source, and be discharged in the condition of carbonic acid or water, and this union must produce as much heat as if the carbon had been burned in oxygen gas. If we suppose that from 2 equivalents of starch 18 equivalents of oxygen are disengaged, and that these 18 equivalents combine with 9 of carbon from the bile, for example, then in this case as much heat must be developed as if the 9 equivalents had been directly burned. Such a phenomenon is analogous to that in the process of fermentation, when by the separation of the elements of sugar into carbonic acid and alcohol, as much heat is evolved as is sufficient to heat every pound of the fermenting liquid by 298°. In like manner in the formation of fat every pound of carbon which obtains the oxygen necessary to convert it into carbonic acid from substances which thereby pass into fat, must disengage as much heat as would raise the temperature of 200 lbs. of water by 70°, or from 32° to 102°. When animals are fattened on food destitute of azote, only certain parts of their structure increase in size; thus, in a goose, the liver enlarges and becomes soft and spongy from the deposition of fat in the cells of that organ. In some diseases the starch, sugar, &c. of the food, do not undergo the changes necessary to fit them for respiration, and consequently to be converted into fat. Thus, in diabetes, the starch is only converted into starch sugar, which is expelled without further change; and in diseases of the liver we find that organ loaded with fat and oil, probably derived from the maltransformation of the bile.

*Food divisible into nutritive and respiratory.* From the previous considerations Liebig infers, that human food consists of two kinds, azotized and non-azotized. The former is adapted to form blood—the latter cannot produce blood. *Nutritive* or *azotized* food consists of vegetable fibrin, vegetable albumen, and casein, animal flesh, and animal blood. In the *respiratory* or *non-azotized* food, are included,—fat, starch, gum, the different kinds of sugar, pectin, wine, beer, and spirits. Now it appears to be a fact well established by recent experiments, that the azotized constituents of food are identical in their composition with that of the solids of the blood; and no azotized body which differs in its constitution from fibrin, albumen, &c. so far as observation goes, is capable of sustaining life. This view of the subject of nutrition gives a most satisfactory explanation of the observations which have been more than once made, and which we recently in this Review took an opportunity of discussing (Brit. and For. Med. Rev., April, 1842—On Food) in reference to the inefficiency of gelatine for the purposes of nutrition. Dogs, when fed on gelatine alone, died from starvation. But when animal flesh was given in conjunction with gelatine, or when vegetable azotized substances were united with gelatine, dogs were sufficiently and properly nourished. It is rather remarkable that no such simple explanation as that presented by Liebig should have offered itself to Magendie, who was sacrificing the inferior animals in multitudes, to satisfy himself of the accuracy of a fact which had been previously settled beyond all question by several individuals, and particularly by Donné. Magendie did not observe the fact, or at least made no use of it, that when gelatine is devoured by dogs, although they are not nourished, yet this entirely disappears, while bone earth alone is found in the excrement. The same observation applies to man when fed on strong gelatinous soup,—not a trace of the gelatine can be detected either in the urine or fæces; it must, consequently, have been consumed for some ultimate purpose in the economy; but what that destination is does not appear so clearly; Liebig conceives it possible that gelatine in the dissolved state may again be converted into cellular tissue, membrane, and cartilage, and may thus serve for the reproduction of such parts of these tissues as have been consumed. In this way he explains the effect of animal jelly in invalids; the organic power by which the constituents of the blood are converted into cellular tissue and membranes, must of necessity be weakened by sickness, and under these circumstances gelatine dissolved, that is, in a form adapted for assimilation, may contribute to strengthen the vital power, as may be done in the case of the stomach by due preparation of general food.

*Changes of the tissues.* It is now satisfactorily ascertained that vegetables produce a substance, termed by chemists *protein*, which constitutes the basis of vegetable albumen; and it appears that out of the protein the various parts of the animal are produced by the vital power. Albumen is that form of protein which seems to constitute the matters from which we may deduce the resulting compounds. All azotized vegetable matters digested by animals must be converted into albumen before they can be endowed with nourishing power. This transformation is produced by chymification in the first instance, or a process which may be aptly enough compared to fermentation, inasmuch as both are changes produced in matter by the contact of another substance. By the action of muriatic acid upon the mucous coat of the stomach, a substance (pepsin) is formed which, by contact with the food, renders it soluble. This phenomenon is quite independent of the vital power, as is proved by the success which attends the experiment when made in vessels out of the body. Lactic acid does not appear to be generated on the healthy human stomach. In the action, therefore, of the fluid of the stomach on food, no other element appears to take a part except the oxygen of the atmosphere and the elements of water. Saliva affords an important supply of water, and also of oxygen, according to Liebig. No fluid possesses in such sufficiency the power of entangling air as saliva. Liebig considers that a large quantity of air gets access to the food on the stomach through the medium of the saliva; and there the oxygen combines with the food, and the nitrogen is given out by the skin and lungs. Rumination in the inferior animals may very possibly have for its object a renewed introduction of oxygen; physiologists have found that nitrogen is given out by the lungs in variable quantities; and Liebig accounts for this by the various proportions introduced into the stomach by the saliva. As a proof that gases can be absorbed into the system from the stomach, he quotes the examples of poisoning by feather white wine, where wine in a state of fermentation has its decomposing condition increased by the temperature of the stomach. The carbonic acid generated is absorbed and penetrates to the pulmonary air-cells, and the patient dies with all the symptoms of asphyxia; the best antidote has been found to be in these cases ammonia. Liebig has proposed some still more ingenious explanations of the source of the excretions. These he finds, by calculation from analyses, to contain all the elements of the blood. In order to form correct views on this subject, he has had the blood analyzed by his well-known plan; not the separate constituents, but a portion of the whole mass: the composition thus deduced he finds to correspond with half the formula of choleic acid (that is, the bile), 1 atom of uric acid, and 1 of ammonia, which is equivalent to saying that the blood ultimately appears in the excretions in the form of bile and urine. This important view is of the utmost consequence to the practice of medicine. In the higher classes of animals, uric acid disappears and is replaced by urea. This substitution obviously depends on the amount of oxygen absorbed in respiration, and also the quantity of water consumed by different animals in a given time. When uric acid is acted on by oxygen, it is converted into alloxan and urea. A new supply of oxygen, acting on the alloxan, changes it either into oxalic acid and urea, into oxaluric acid, or into carbonic acid and urea. The mulberry or oxalate of lime calculi are usually found in those in whom, from want of exercise, the supply of oxygen has been deficient. Calculi, containing uric acid, or oxalic acid, are never found in consumptive patients; and it has been remarked in France that when patients, affected with uric acid calculi, are removed for exercise to the country, they become subject to the mulberry calculus. It is erroneous to suppose that the mode of cooking food can have any influence on these diseases. Flesh, in whatever way prepared, is at once converted into blood, while the uric acid and urea are derived from the transformed tissues. If this azotized food is properly supplied, and if an equivalent amount of oxygen is afforded to the system, none of these concretions can occur; in other words, if the liver and kidneys are capable of transforming the tissues which are continually undergoing change into urea, uric acid, and bile, none of these concretions should occur. Uric acid calculi have never been observed in carnivorous animals in the wild state; and it is believed that gravel and calculus occur in persons who use very little animal food. In reference to the bile, Liebig considers it to be derived from the decomposition of protein and starch; and he infers that, if the elements of protein and starch, oxygen and water being also present, undergo transformation together, and mutually affect each other, we procure, as the results of this transformation, urea, choleic acid (or bile), ammonia, and carbonic acid, and no other product besides these whatever. The metamorphosis of the compounds of protein present in the body is effected by means of the oxygen carried by the arterial blood, and of the elements of starch rendered soluble in the stomach, and carried to every part to enter into the newly-formed compounds. We have presented to us, the principal constituents of the animal excretions and secretions; carbonic acid, the excretion of the lungs,—urea and carbonate of ammonia,—expelled by the kidneys, and choleic acid separated by the liver.

Some of the speculations of Professor Liebig, relative to the action of vegetable alkalies on the system, are highly curious: all the azotized alkalies, with the exception of three or four, are poisonous. Caffein, the alkaloid of tea and coffee, is not poisonous; and this substance, by the addition of 9 atoms of water and 9 atoms of oxygen, may be resolved into 2 atoms of taurine, a substance procured from choleic acid (the bile) by the action of muriatic acid. This substance, like the medicinal alkaloids, may, from the similarity of its composition to the brain and nerves, combine with them and supply their waste. There is nothing so absurd in this as some might be inclined to infer; for we must remember that the animal organism has produced the brain and nerves out of compounds furnished by vegetables. If then we grant that the brain and nerves are formed from vegetable albumen, is it unreasonable to conclude that substances (vegetable alkalies), intermediate in composition between the fats and the compounds of protein, may be employed in the organism for the same purpose?

In the foregoing report, we have confined ourselves to a scanty analysis of some of the most important of the new views of Liebig. We do not affirm that all these views are demonstrably correct; but we believe that he has assumed the proper ground for physiological research; that in short all his speculations are in the proper direction. His book when it appears will present a rich mine of ideas, which we have no doubt will be purloined and diluted after various fashions. We trust that the present outline will preserve some of the property to its proper owner; and that those who build other works, by assuming some of his ideas, and surrounding them by a multitude of their own common-places will, unlike the plagiarists who have borrowed from his work on *agriculture*, have the honesty to acknowledge the source of their materials. At another opportunity, we shall present to our readers Liebig's views respecting the mechanical movements.

GIESSEN; *June* 1, 1842.

